# The New Reform of the National Health System in Morocco: An Opportunity to Meet the Challenges and Improve the Practice of Anesthesiology

**DOI:** 10.7759/cureus.57841

**Published:** 2024-04-08

**Authors:** Wafaa Harfaoui, Lahcen Belyamani, Aziz Zentar, Brahim Lekehal, Majdouline Obtel

**Affiliations:** 1 Epidemiology and Public Health, Laboratory of Community Health, Preventive Medicine and Hygiene, Faculty of Medicine and Pharmacy, Mohammed V University in Rabat, Rabat, MAR; 2 Epidemiology and Public Health, Laboratory of Biostatistics, Clinical Research and Epidemiology, Faculty of Medicine and Pharmacy, Mohammed V University in Rabat, Rabat, MAR; 3 Mohammed VI Foundation of Health Sciences, Mohammed VI University in Rabat, Rabat, MAR; 4 Royal Medical Clinic, Mohammed V Military Hospital in Rabat, Rabat, MAR; 5 Faculty of Medicine and Pharmacy, Mohammed V University in Rabat, Rabat, MAR; 6 Direction, Military Nursing School of Rabat, Rabat, MAR; 7 General Surgery, Mohammed V Military Hospital in Rabat, Rabat, MAR; 8 Vascular Surgery, Ibn Sina University Hospital Center, Rabat, MAR

**Keywords:** anesthesia, moroccan health system reform, access to care, service quality, patient safety

## Abstract

The health sector in Morocco has undergone major changes in recent years, thanks to the reform of the national health system. This reform aims to improve accessibility and the equitable distribution of care throughout the country and enhance the quality and safety of health services.

Anesthesia plays an important role in improving patients' quality of life and reducing the risks associated with surgical procedures. However, anesthetic practice in Morocco faces several challenges, including territorial disparities, unequal access to anesthetic services, financial constraints, a shortage of qualified staff, insufficient continuing education, and the need for appropriate administrative and legal frameworks regarding current anesthesiology practice.

The reform of the Moroccan national health system has the potential to significantly advance the practice of anesthesia in Morocco. Indeed, this reform includes a number of measures that could help to improve access to anesthesia services, enhance the quality of care, ensure patient safety, and promote research in this field. Its success will depend on the effective implementation of planned measures and the ability to overcome potential obstacles.

## Introduction and background

The Moroccan healthcare system, despite its creation in 1959, has not yet achieved its full potential for sustainable transformation. Its development has been hampered by major challenges such as inequalities in access to care, staff shortages, and deficiencies in governance. These obstacles have hindered the emergence of a high-performance healthcare system that is accessible to all.

However, in recent years, the health sector in Morocco has undergone significant transformation. These changes should continue in the future due to decisions and actions taken as part of the government's 2021-2026 program [1], the new development model 2021 [2], as well as Framework Law 09.21 [3] on social protection. This initiated the bill to overhaul the national health system, set out in Framework Law 06.22 [4], which aims to create a new, resilient, and harmonized system that is capable of supporting the major initiatives undertaken. This reform of the national health system aims to improve access to health services, enhance their quality and safety, and ensure the equitable distribution of care across the country.

The changes brought about by the reform of the national health system in Morocco may have an impact on multiple medical fields, including anesthesiology, which plays an essential role in modern healthcare by ensuring the safety and quality of care, facilitating surgical procedures, and managing pain for patients [5]. Anesthesiology, as a specialty, has made extraordinary progress concerning anesthetic safety [6] through the effective combination of training advancements, newer drugs, cutting-edge technology, and the adoption of guidelines and safety checklists in clinical practice [7]. In low-income countries, progress in anesthesiology is limited by poor infrastructure, staff shortages, drugs, equipment, and supplies. As a result, standards of anesthesia care are often lower in countries with a low human development index than in those with a high index [8,9]. The Institute of Medicine has described quality healthcare as safe, timely, effective, efficient, equitable, and patient-centered [10], and the World Health Assembly recognizes that access to essential anesthesia and emergency and surgical care is an integral part of universal health coverage (UHC) [11]. It is important to know that anesthesia plays an adjuvant role in the therapeutic process and carries inherently high risks that require rigorous management. The mission of the Anesthesia Patient Safety Foundation is to ensure that all patients undergoing anesthesia are protected from harm [12].

In Morocco, anesthesiology has undergone specific development in recent years as a result of better training being delivered to anesthetists, the use of new anesthetic drugs, and improved perioperative monitoring [13,14]. The creation of the Moroccan Society of Anesthesia, Analgesia, and Critical Care in 1984 [15] was a major event in this development. However, despite the progress made in the field of anesthesia in Morocco, the country still faces several major challenges that must be addressed to improve patient care and outcomes, as there is a significant gap between current practice and the recommended standards.

Among the challenges to be overcome are major territorial disparities, which have repercussions in the field of anesthesia. Access to high-quality anesthetic care is strongly influenced by the unequal distribution of healthcare services and socio-economic factors, to the detriment of the poorest patients. In addition, the lack of qualified human resources is a major obstacle. This lack is the result of several interdependent factors, including an insufficient number of university courses in anesthesiology, the emigration of anesthetists, and the limitations of continuing training. There are also financial constraints resulting from the low budget allocated to the Ministry of Health and Social Protection (MHSP), limited investments in the field [16], and the need for a clear and appropriate administrative and legal framework for the current practice of anesthesiology in order to guarantee patient safety. These factors can affect the quality of care provided. In addition, a comprehensive root cause analysis is needed to identify all factors likely to affect the quality of care and patient safety in anesthesia and to implement corrective solutions adapted to real and current conditions.

The aim of this literature review is to analyze the impact of reform in the Moroccan healthcare system on the practice of anesthesia. The study examines how these reforms can improve access to anesthesia services, enhance the quality of care, guarantee patient safety, and promote research in this field.

To this end, a brief history of previous administrative reforms will be undertaken in order to demonstrate the progression of reforms in the health sector in Morocco, and the main thrusts of the new reform will be analyzed. Next, the specific challenges facing anesthesia in the current context will be identified using national statistical data and government documents relating to the reform. Furthermore, the impact of the measures taken by the reform on access, quality, and safety of anesthetic care will be assessed.

Finally, the future prospects for anesthesia in Morocco will be explored, taking into account international trends and the implications of the reform. By meeting the challenges and capitalizing on the opportunities offered by the reform, Morocco has the potential to significantly advance the practice of anesthesia. The success of the reform will depend on the effective implementation of the planned measures and the ability to overcome potential obstacles.

## Review

The main thrusts of the new reform of the health system in Morocco

Over the decades, Morocco's healthcare system has undergone significant changes in response to various factors, such as territorial disparities, inequalities in access to care, a shortage of healthcare professionals, governance problems, and insufficient funding. The Moroccan government has introduced a number of reforms and initiatives aimed at remedying these problems and improving access to healthcare for all citizens. The ultimate aim of these reforms is to ensure UHC and to build a more efficient and effective health system that meets the needs of the population.

Table 1 below presents a chronology of the evolution of the health system in Morocco, highlighting the key periods, reforms, and initiatives undertaken by the government to advance the country's health system.

**Table 1:** Evolution of the health system in Morocco: chronology of reforms and initiatives (1959–2024) BMC: Basic Medical Coverage; MAP: Medical Assistance Plan; SDGs: Sustainable Development Goals; MHSP: Ministry of Health and Social Protection; CHI: Compulsory Health Insurance; HAH: High Authority for Health; THG: Territorial Health Groups; MAMHP: Moroccan Agency for Medicines and Health Products

Period	Event	Description
Period (1959-1980)	A period of foundation and consolidation for the Moroccan national health system	The Moroccan national health system was established in 1959. The first national health conference was organized in April 1959 under the presidency of His Majesty Mohamed V, with the principles set out in the declarations that the health of the nation is the responsibility of the state and that the Ministry of Public Health must ensure its conception and realization [17]. The system gradually evolved through infrastructure creation, resource nationalization, and efforts against epidemics. Five development plans were implemented to nationalize and improve healthcare [17]. Key milestones included the establishment of medical faculties and vocational training schools. In 1976, the communal charter delegated healthcare responsibilities to local authorities [17]. Morocco signed the Alma-Ata Declaration in 1978 [18] and enacted a decree in 1980 to organize health coverage and services. These principles guided economic and social development plans until 1980 [18].
Period (1981-1995)	The evolution of the Moroccan healthcare system and its commitment to primary healthcare	In the global excitement around the importance of primary health care, Morocco focused on extending primary health care (in the 1980s and 1990s) [19]. Morocco has demonstrated its commitment to the Declaration of Alma Ata and primary healthcare through various initiatives. From 1981 to 1985, the country strengthened its basic health structures and developed health programs as part of a five-year plan [18]. In 1986, the adoption of the Ottawa Charter demonstrated Morocco's commitment to health promotion. In 1989, a study on the financing of healthcare was carried out, reflecting the growing concerns of the Moroccan health authorities [20]. From 1990 to 1998, the projet d'investissement dans le secteur de la santé in Morocco aimed to re-establish sustainable nationwide health programs, strengthen primary care, and improve the administration of health services [21]. In 1994, the Moroccan Ministry of Health undertook a major restructuring, marking the beginning of hospital reform, and several changes were introduced, such as the creation of new central directorates for hospitals, medicines, and regulations [22]. This restructuring took place in 1995 [20] and played a fundamental role in laying the foundations for subsequent reforms, in particular the BMC.
Period (1996- 2010)	Aspiration for renewal and announcement of a transformation	During this period from 1996 to 2002, Morocco has undertaken major reforms in the field of health, in particular, the reform of regionalization and financing [23]. The country has fostered new partnerships and collaborations, focusing on key aspects such as regionalization, hospital reform, and improved health insurance [24]. Between 1999 and 2006, the government set up two major projects in the health sector. The first project, the Health Sector Management Financing Project, aimed to improve the management of public hospitals, the quality of care, and the financing of the health sector. Concurrently, the Health Sector Management Support Project aimed to improve the performance of the health sector in the Oriental region [21]. In 2000, the country adopted the Millennium Development Goals (MDGs) and put in place health strategies focusing on regionalization, hospital reform, and improved financial management [23]. In 2002, the promulgation of Law 65-00 marked the implementation of the reform of BMC [25], giving rise to the BMC and the basic MAP. In 2005, the National Initiative for Human Development was launched [26]. In 2008, MAP was implemented [27], and a health action plan was drawn up for the period 2008-2012 [28]. From 2005 to 2010, the support program for regionalization, deconcentration, and strengthening of basic healthcare was launched to strengthen primary healthcare and management capacities at provincial and regional levels, as well as to support administrative and budgetary reforms [21].
Period (2011-2019)	Development of the right to health and reform of the health system	In 2011, a new constitution was adopted, recognizing the right to healthcare and medical coverage [29]. In the same year, Framework Law 34-09 was promulgated, establishing the fundamental principles and objectives of the state's action in the field of health and organizing the health system [30]. In 2012, the country launched the MAP to provide medical cover for vulnerable populations [18]. Between 2012 and 2016, a health sector strategy was implemented based on human rights and health democracy [18]. In 2013, a second national health conference brought together stakeholders in the healthcare system [17]. In 2015, Morocco made a commitment to the international community to achieve the SDGs [23]. Two Health Sector Reform Support Programme projects were launched in the periods (2009-2015) and (2015-2018), respectively, to ensure equitable access and improve the quality of care. The program for results (2015-2019) has also been set up to strengthen access to primary care in rural areas and improve the governance of the healthcare system [21]. In 2019, the country mobilized against the COVID-19 pandemic and is planning a reform of the healthcare system in conjunction with the generalization of social protection [31].
Period (2020-2024)	Toward a new reform and a more efficient and equitable healthcare system	In 2020, the royal speech called for an overhaul of the national healthcare system to anticipate future challenges [32]. In 2021, Framework Law 09-21 was adopted, allowing the generalization of social security coverage and extending CHI even for MAP beneficiaries [3]. In 2022, the new reform 06.22 was implemented [4]. In 2023, the creation of THG [33] was created to reorganize healthcare provision, followed by the creation of the HAH to guarantee the continuity of healthcare services and supervise health insurance [34]. Also in 2023, the MHSP was reorganized with the creation of the MAMHP [35], the Moroccan Agency for Blood and Blood Derivatives [36], and the Healthcare Employment [37]. These measures aim to improve the quality and efficiency of the Moroccan healthcare system.

Currently, the new reform of the health system in Morocco, backed by Framework Law 06.22, is an ambitious project that aims to remedy dysfunction in the sector, in particular, the succession of several reforms without bringing about any real change. The Framework Law 06.22 is divided into eleven chapters covering various aspects of the healthcare system, such as general points, the rights and duties of the population, healthcare provision, healthcare establishments, the national health map and regional health maps, public-private partnerships, human resources, training, research, and innovation in the healthcare field, digitization of the healthcare system, the accreditation system for healthcare establishments, and management and governance bodies. Finally, there are the final proposals. This Framework Law will be implemented by means of subsequent legislative and regulatory texts, and it cancels and replaces Law 34.09 [30] on the health system and healthcare provision.

The new reform was initiated by Framework Law 09.21 on social protection, which provides for the extension of social cover to all Moroccans by 2025 and comprises four components: the extension of Compulsory Health Insurance (CHI), family allowances, retirement pensions, and compensation for loss of employment. These measures should make it easier for citizens to access health services, improve the quality and safety of these services, and ensure that care is evenly and fairly distributed throughout the country. The objectives of the new reform of Framework Law 06.22 are based on four pillars: good governance, human resources development, the upgrading of healthcare provision, and digitalization.

Firstly, in accordance with Article 32 of Framework Law 06.22, the adoption of good governance is based on the creation of management and governance bodies aimed at strengthening the regulation of the actions of those involved, consolidating hospital governance, and the territorial planning of healthcare provision through the creation of the High Authority for Health (HAH) under Law 07.22 [34]. It is a public legal entity with financial autonomy that ensures the continuity of the state's action in the field of health and provides its opinion on public policies in the field of health. Its responsibilities include providing technical supervision for compulsory basic health insurance and ensuring the preparation of control measures for its system. This involves evaluating the quality of services provided in public and private healthcare facilities and assessing the condition of patient medical care. Additionally, it involves accrediting public and private healthcare establishments based on national indicators, standards, and references determined by the authority. The role also entails monitoring, analyzing, and evaluating epidemiological data, as well as evaluating disease prevention programs. Furthermore, it conducts studies and research within its area of expertise, either independently or at the request of the government or parliament. Developing guidelines and references related to the continuous training of healthcare executives is another responsibility. Lastly, within its jurisdiction, the position involves mediating in disputes brought before it by healthcare professionals.

The creation of Territorial Health Groups (THG) under Law 08.22 [33] in the form of public establishments responsible at the regional level for the implementation of the state's health policy. Each group includes all public health facilities located within its territorial jurisdiction and aims to resolve the challenges and obstacles facing healthcare provision at the regional level. This initiative is based on the recommendations of a strategy aimed at merging university hospital centers (CHUs) and regional hospital units into a single autonomous public establishment responsible for healthcare, training, and scientific research. The aim is to optimize public healthcare provision at the regional level. According to the law, each region must set up a THG according to a regulatory text that will also specify the roles and responsibilities of each THG, dividing its tasks into six key areas: healthcare provision, public health, care, training, research and innovation, and administration. The creation of these THGs is an important step in the reform of the Moroccan healthcare system; they will enable better coordination of the activities of the various actors in the healthcare system at the regional level to improve efficiency, equity, and accessibility of care.

The creation of the Moroccan Agency for Medicines and Health Products (MAMHP) under Law 10.22 [35], which is responsible for coordinating national pharmaceutical policy, regulating the pharmaceutical sector, and ensuring the availability, safety, quality, and access to medicines and healthcare products, Additionally, the creation of the Moroccan Agency for Blood and Blood Products (MABBP) under Law 11.22 [36] aims to develop blood stocks capable of meeting national needs and to guarantee the availability, safety, and quality of all blood-derived products, whatever the circumstances. These agencies will contribute to enhancing the country's health security and improving access to healthcare services.

Second, the development of human resources in accordance with Chapter VII of Framework Law 06.22 [4] and through the promulgation of Law 09.22 [37] on the healthcare professions, aimed at motivating healthcare professionals and offering them a clear and attractive status, guaranteeing them protection, dictating the implementation of continuous training sessions and programs throughout their professional career, and providing for an efficient and motivating remuneration system for healthcare professionals and promising career prospects and satisfactory working conditions, human resources management is essential to the smooth running of an organization. Motivating human resources is a key success factor. This motivation is focused on organizational efficiency and takes into account the personal and professional development of human resources. The reform also aims to reduce staff shortages by attracting new healthcare professionals, creating new positions, and encouraging Moroccan healthcare professionals living abroad to return to practice in their country of birth.

Thirdly, the implementation of healthcare provision in accordance with Chapter III of Framework Law 06.22 [4], which stipulates that healthcare provision includes human resources, public and private health infrastructures, and the means to provide care and health services. The state is responsible for ensuring the balanced and equitable distribution of healthcare provision throughout the country. The public and private sectors must work synergistically to respond effectively to healthcare needs. Healthcare provision is organized at the regional level, in accordance with the regional health map, and by promoting the care pathway. The state is also committed to improving public healthcare infrastructure and attracting national and foreign skills and foreign investment to develop healthcare provision and improve the quality of healthcare services.

The last point, the commitment to the process of digitizing the healthcare sector, as described in Chapter VIII of Framework Law 06.22, includes the implementation of an integrated national health information system (HIS) to monitor and evaluate the performance of the healthcare system. In accordance with Article 28, this system will collect and process all data concerning public and private healthcare establishments, their activities, and their resources.

In addition, Article 29 provides for the creation of an integrated IT system, known as the "shared medical record," within the information system mentioned in Article 28, to identify, monitor, and evaluate each patient's care pathway in compliance with the legislative and regulatory provisions in force regarding the protection of personal data.

The challenges of anaesthetic practice in Morocco

The Challenges of Accessibility

Territorial disparities and inter-regional inequalities in economic growth persist in Morocco, with differences linked to economic growth, social sectors, and economic dynamics. The more developed regions offer better access to basic social services, such as education, health, and sanitation, while the less-developed regions suffer from a lack of infrastructure and social services [38,39,40]. These inequalities are also present between regions of the same country, with disparities in the living environment of inhabitants and socio-economic changes. The determining factors in the evolution of these disparities are urbanization, migration, and the concentration of national wealth in specific regions.

The existing studies highlight the importance of combating economic, social, and territorial inequalities and correcting existing disparities. This infers putting in place programs and strategies to reduce these inequalities, as well as actions to promote social justice and good governance. Territorial and inter-regional disparities persist in Morocco, which impact economic growth, social sectors, and economic dynamics [41]. Combating these inequalities requires targeted action to strengthen social and territorial cohesion.

Access to healthcare in Morocco is marked by profound inequalities that manifest themselves at several levels, particularly between regions and between urban and rural areas. People in the least developed regions, who often face more precarious socioeconomic situations, have limited access to healthcare, including anesthesia services. The cesarean section rate is higher in urban areas (26.3%) than in rural areas (12.9%), and there are only eight emergency medical services in the whole of Morocco. The Rabat-Salé-Kénitra region has 21 mobile emergency and resuscitation services (MERS), compared with just one MERS in the Beni Mellal-Khénifra region [42]. Similarly, the Fès-Meknès region has 190 units of basic emergency transport equipment, compared with the 39 units only for the Beni Mellal-Khénifra region. According to data from the MHSP, Morocco has only 165 hospitals [42]. The distribution of these establishments varies between regions and is primarily concentrated in the most densely populated areas; for example, there are 27 hospitals in the Casablanca-Settat region, compared with five in the Guelmim-Oued region. The hospitals in Morocco have a combined functional capacity of 20,364 beds. The number of surgical operations was 270,723 in 2021, compared with 2,343,399 in 2020 [43]. This number is increasing but remains lower than in other developed countries, with 143 surgical operations per doctor and an average length of hospital stay of 4.3 days. Morocco has 384 healthcare clinics [42]. The distribution of these clinics is also uneven; again, they are concentrated primarily in the most densely populated regions. The Casablanca-Settat region alone has 117 clinics, while the Drâa-Tafilalet region has four [42]. Even more alarmingly, specialists are mostly concentrated on the Casablanca-Rabat-Marrakech-Fez axis [44], and this staff density has not benefited the different regions of the kingdom equally. Furthermore, the criteria on which the distribution of healthcare professionals between care networks, settings, and establishments is based are subjective. As a result, there are geographical disparities, imbalances in population coverage, and mismatches between care structures and the human resources allocated to them. In addition, there is underutilization of the available human resources [45]. The Casablanca-Settat region has 3,400 medical health workers and 6,335 paramedical health workers, while the Dakhla-Oued Ed-Dahab region has only 72 and 295, respectively [44].

The Challenges of Quality and Safety of Care in Administering Anesthesia

Lack of financial resources: In Morocco, insufficient funding for the health sector remains a major challenge, despite efforts made. Currently, only between 6% and 7% of the general state budget is devoted to this crucial area, which is well below the recommendations of the WHO (12%) and the African Union's Abuja Declaration (15%) [46]. According to the Numbeo Healthcare Index by Country 2024, Morocco's healthcare system is ranked 7th in Africa and 92nd worldwide [47].

Although the draft Finance Law for 2024 provides for an increase in the budget allocated to the Ministry of Health, from MAD28.13 billion in 2023 to MAD30.7 billion in 2024, an increase of approximately MAD2.56 billion (+9.1%) [48], this increase remains insufficient to fully meet the needs of the health sector.

It is essential to emphasize that health is a critical area that requires adequate funding to guarantee quality access to healthcare for all citizens. A significant and sustainable increase in the budget allocated to health is needed to strengthen health infrastructure, improve healthcare services, recruit and train qualified medical staff, and put in place effective prevention programs.

Severe shortage of anesthesiologist-intensivist physicians: According to the Ministry of Health, Morocco currently has 196 anesthesiologist-intensivist physicians in the public sector, compared with 222 during the COVID-19 period, while 462 others work in the private sector, yielding a total of 658 anesthesiologist-intensivists [49]. Furthermore, the majority of anesthesiologists-intensivists are concentrated in large cities, notably 213 in Casablanca, 88 in Rabat, and 44 in Marrakech. Furthermore, there are currently 297 physicians in training [49].

However, the WHO, the World Federation of Societies of Anaesthesiologists (WFSA), and the Lancet Commission on Global Surgery have set an ambitious target of having at least 20 surgeons, anesthetists, and obstetricians per 100,000 population by 2030. This target is crucial to ensuring the safety of anesthesia delivery worldwide [50].

Among the causes of the shortage of anesthesiologist-intensivists in Morocco is the so-called “brain drain." A study has shown that 13.5% of Moroccan anesthesiologist-intensivists are currently working abroad. Of these, 4.5% are in France, and 3% are in Germany and Spain. There are many reasons for this professional emigration. Some Moroccan anesthesiologist-intensivists are motivated by the search for a better quality of life, often combined with better working conditions and remuneration abroad. For others, the desire for professional fulfillment and access to cutting-edge technology plays an important role. Finally, personal factors such as family reunification can also motivate emigration [51].

In Western countries, anesthesiology is a singular specialty [52], while intensive care provision is another [53]. Accordingly, these are considered two different specialties; however, the physician in anesthesiology and intensive care fulfills both roles in the Moroccan healthcare system [54]. The anesthesiology and intensive care medicine professions in Morocco are not very attractive. One study showed that the selection of anesthesiology as a specialty is influenced by several crucial factors, such as the length of training, intellectual interest, the quality of the relationship between doctor and patient, and financial aspects. However, other factors may discourage students from choosing this specialty, including a lack of mentorship, an excessive workload, and not enough time to spend with family [55]. According to a study conducted by the University of Medicine and Pharmacy in Marrakech, only 7% of doctors completing their training chose to specialize in anesthesiology [56].

Specialty in anesthesiology and intensive care nursing: a profession requiring an appropriate and precise administrative and legal framework: According to the recommendations, the nurse/patient ratio should be at least two nurses for every five patients in intensive care, 24 hours a day [57]. However, the nurse anesthetist and intensive care ratio has not been calculated, making the number of anesthetists available in each country uneven. For example, in high-income countries, there were 54,661 nurse anesthetists for a population of 331.6 million in the USA and 10,648 nurse anesthetists for a population of 66.8 million in France [58]. This contrasts sharply with low-income countries, such as Morocco, where there were 2,394 nurse anesthetists in the public sector for a population of 36,67 million inhabitants [44].

It is important to note that nurse anesthetists play an important role in the provision of high-quality anesthesia care and are considered essential members of the anesthesia care team. However, the legislative texts governing the specialty of anesthesiology in Morocco, particularly Article 2 of Ministerial Decree No. 2150-18 establishing the list of duties assigned to officials belonging to the interministerial body of nurses and healthcare executives [59], and Article 6 of Law No. 43-13 stating that the nurse anesthetist and intensive care nurse perform anesthesia and resuscitation procedures on patients under the supervision and direct surveillance of an anesthesiologist-intensivist [60]. However, its implementation is currently the subject of debate as to its feasibility in the current context of anesthesiologist shortages and its implications for anesthesia patient safety.

Due to the shortage of anesthesiologist-intensivist physicians, some hospital administrations have authorized nurse anesthetists to perform anesthesia procedures without the supervision of an anesthesiologist. This decision has sparked reactions from trade unions and the Moroccan Association of Nurses in Anesthesiology and Intensive Care [61], who have expressed concerns about potential confusion and legal issues that may arise. They are calling for solutions to be found.

To ensure the continuity of anesthesia care for Moroccan citizens, the Ministry of Public Health and Social Protection has implemented temporary measures to address the absence of anesthesiologist-intensivists. In a circular, the Minister requests that nurse anesthetists and resuscitators in the public sector temporarily perform emergency interventions prescribed by the surgeon or the responsible emergency physician that cannot be postponed in the absence of a specialist in anesthesia and resuscitation. The Minister clarifies that the provisions of Law 43.13 have not yet been enforced, as the necessary implementing regulations for their full application have not yet been published. Thus, the Minister emphasizes the importance of guaranteeing the right to life and health for all citizens in accordance with Article 20 of the Constitution, noting that the refusal to provide assistance to a sick or endangered person constitutes an offense punishable by the penal code, and the punishment is much more severe than the civil liability arising from anesthesia procedures [62].

The circular has elicited responses from the Moroccan National Federation of Anesthesiology and Reanimation in Morocco [62,63], and the Moroccan Society of Anesthesiology, Analgesia, and Critical Care, who have underscored the importance of patient safety protocols and emphasized that national and international guidelines explicitly state that anesthesia-resuscitation is a medical procedure that should be conducted in the presence of a specialized physician. They highlight the essential role of anesthesiologists and resuscitators in ensuring the quality and safety of anesthesia for patients and the smooth progression of the anesthesia process.

This situation highlights the challenges faced by anesthesia in Morocco, such as the shortage of qualified healthcare professionals in anesthesia and the need for clear and precise administrative and legal frameworks that are adapted to the current context. In the face of these challenges, it is imperative to find sustainable and radical solutions. The training of an increased number of anesthesiologist-intensivists is essential to addressing the personnel deficit. Furthermore, improving working conditions, both in terms of resources and financial aspects, is necessary to attract and retain professionals in this critical field.

Inadequate training and professional development: Moroccan public hospitals are required, in collaboration with development training establishments, to carry out training and scientific research activities in public health, health economics, and health administration as part of the national health system, according to Article 2 of the Decree on Hospital Organization [64] and Article 8 of Law 09. 22, which stipulates that local and regional authorities must offer healthcare professionals, throughout their careers, training aimed at developing their skills and qualifications and to constantly improve the quality of their services [37]. However, the training system in public hospitals is often considered to be unsatisfactory and poorly adapted to the real needs of staff. Several factors contribute to this situation, one of the most important being the lack of funding allocated to training. In Morocco, the budget allocated to training, research, and teaching is notoriously inadequate, representing less than 2% [65].

Measures taken by the Moroccan government to improve the health system and its impact on anesthesia

The reform of the Moroccan healthcare system is an important initiative that aims to have a significant effect on the accessibility, quality, and safety of care across all medical fields, including anesthesiology, through the adoption of good governance, which is a key factor in improving health outcomes. This approach enables governments to manage their human and financial resources effectively and to provide quality healthcare services to all citizens [66].

To improve access to quality healthcare for all, the World Bank has approved a 450 million US dollar loan to support the reform of the Moroccan healthcare system. This loan will lay the foundations for an inclusive and high-quality healthcare system by expanding coverage in underserved areas, improving access to quality healthcare services, strengthening the healthcare workforce, and enhancing the governance of the healthcare system [67].

Morocco is taking ambitious steps to reform its healthcare system and improve access to healthcare for all. These measures include building and renovating hospitals, digitizing the healthcare system, increasing the budget allocated to the health sector, training and bolstering healthcare staff, and improving continuing and professional training.

Impact of the New Reform on Access to Anesthesia

The reform of the national healthcare system aims to improve access to healthcare and anesthetic care through several actions.

Generalizing medical cover: According to the WHO, UHC means that everyone, regardless of socioeconomic status, has access to the health services they need without the cost of causing financial hardship for users. This concerns all essential health services throughout life, from health promotion to prevention, treatment, rehabilitation, and palliative care [68]. This provisioning aims to achieve target 3.8 of the Sustainable Development Goal (SDG) 3 by 2030, set by the United Nations in 2015. This goal aims to ensure that everyone has UHC, including protection against financial risks and access to healthcare services, essential medicines, and vaccines that are safe, effective, of good quality, and affordable [69]. The WFSA emphasizes the importance of anesthesia as part of UHC and is committed to taking the lead in achieving universal access to safe anesthesia through the publication of its official position statement on anesthesiology and UHC [70].

Moroccan Framework Law 09.21 expanded CHI to 22 million people, achieving nearly 90% coverage by 2023. This program aims to generalize family allowances to include 7 million school-aged children (operational during 2023-2024), put in place operational procedures to generalize the social assistance system by 2024, extend membership in pension schemes to 5 million people by 2025, and generalize compensation for loss of employment by 2025 [3]. To ensure the effective targeting of the beneficiaries of various social protection programs, in 2023, the state generalized and deployed the National Population Register (NPR) and the Unified Social Register (USR) in all of the kingdom's provinces and prefectures. These databases collect information on Moroccan citizens to guarantee easy access to social assistance programs and improve their effectiveness. An assessment of enrolment up to September 27, 2023, shows that more than 13.6 million people were enrolled in the NPR and 2.8 million households, equivalent to 9.7 million people enrolled in the USR [48].

On the other side, the MHSP, the Mohammed V Foundation for Solidarity, and MEDIOT Technology have signed a partnership agreement aimed at improving access to healthcare in rural areas. The program provides for the deployment of connected mobile medical units that will offer basic healthcare services to people living in these regions. The aim is to improve access to healthcare for people living in rural areas who suffer from a lack of access to healthcare services. The program includes doctors, nurses, and administrative assistants and is equipped with cutting-edge biomedical equipment that enables face-to-face medical consultations and specialist teleconsultations to be carried out. A budget of MAD180 million has been committed to its creation [71].

Strengthening the governance of the national healthcare system: The new reform of the Moroccan healthcare system, enacted by Framework Law 06.22, includes a number of important measures, particularly the introduction of new governance, the creation of management and governance bodies, and the adoption of laws and regulations.

The THG, created by Law 08.22, is a regional steering tool for reorganizing healthcare provision and optimizing the availability and pooling of resources. The reform provides for the creation of the HAH by Law 07.22, which aims to ensure the continuity of healthcare services provided by the state. It is responsible for overseeing health insurance, evaluating the quality of healthcare services, and influencing national policies in the field of healthcare. The MHSP reorganized its organizational chart by creating two new agencies: the MAMHP by Law 10.22 and the MABBP by Law 11.22. The role of these agencies is to guarantee Morocco's health sovereignty by ensuring the availability, quality, and safety of medicines and blood products.

Improving and implementing healthcare provision and infrastructure: The WHO recommends that countries construct and modernize hospitals, train and recruit more healthcare professionals, and invest in high-quality equipment and medicines. It stresses the importance of strengthening health infrastructure, preparedness, response, and recovery capacities for health security while also urging investment in essential public health functions [72]. Several research studies confirm the importance of investing in hospitals and infrastructure for improvements in anesthesia processes [73,74]. The MHSP has invested MAD3 billion in the purchase of medical and hospital equipment, including more than 1,000 intensive care beds and 550 respirators, as well as the purchase of medicines, including pharmaceutical products, medical consumables, and medical gases [75]. It has also launched an ambitious program to extend and renovate hospital services to increase the bed capacity of health establishments and improve the care on offer. Extending and upgrading hospital services and increasing hospital bed capacity by more than 2,000 additional beds between 2021 and 2023 has enabled the commissioning of several new hospital establishments [48].

MHSP Protection has drawn up a program with university hospitals to modernize the infrastructure and equipment of level three hospitals. The rehabilitation and equipment development work will take place over two years and is expected to cost MAD1.7 billion. The five and three-level hospitals covered by this agreement are the Fes, Casablanca, Rabat, Marrakech, and Oujda CHUs [76].

A total of 590 rehabilitation operations, 369 programmed construction operations, and the acquisition of 735 mobility units have been updated for the period of 2017-2023 [76].

In the pharmaceutical sector, the MHSP has made significant progress in improving access to medicines and health products. The national pharmaceutical industry now covers more than 70% of the needs of the local market, thanks to the promotion of the local manufacturing of medicines for chronic and costly diseases. This has helped to improve the rate of use of generic medicines, which currently stands at 40% [76].

Digitalization: The electronic patient record (EPR) can play a key role in improving the quality of care services, provided these are fully integrated into the work processes of healthcare professionals. This incorporation offers a number of advantages, such as facilitating communication between the various parties involved in patient care, guaranteeing the traceability of care, and facilitating access to medical information. When used appropriately, the EPR can therefore encourage ongoing improvement in professional practices [77]. The MHSP has launched a new HIS, estimated to cost MAD1.2 billion, aimed at modernizing the Moroccan healthcare system [48].

Impact of the New Reform on Anesthesia Quality and Safety

Increasing the health budget: In line with the recommendations of the WHO and the African Union on healthcare financing, the recent increase in the budget of the Moroccan MHSP is expected to have several positive implications for healthcare quality and will allow investment in the construction of new hospitals and healthcare centers, the acquisition of new medical equipment, the training of health personnel, and the promotion of medical research to improve the quality and safety of surgical and anesthetic care [69].

Based on the above, the budget of the MHSP has risen sharply, from MAD6.1 billion in 2006 to MAD30.7 billion in 2024, a five-fold increase [78] that reflects the Moroccan government's commitment to improving the sector and ensuring the accessibility, quality, and safety of healthcare for all citizens. The MHSP's budget has been allocated into two main categories: management and miscellaneous expenditures, and the investment in and strengthening of healthcare infrastructure to support the sector's current projects. The government has also allocated additional funding to the special central pharmacy account to ensure the availability of essential medicines [76].

Development of human resources: The WHO recommends the availability of a sufficient number of qualified healthcare professionals to meet patients' needs and ensure the quality and safety of anesthetic care [69]. Chapter VII of Framework Law 06.22 and Law 09.22, relating to healthcare professions, introduced a remuneration system for motivating healthcare professionals. The implementation of continuous training and recruitment of new anesthetists, as well as the creation of new posts, should help to reduce the shortage of anesthetists at the national level by attracting and retaining these professionals.

The adoption of two laws, Law 33.21 [79], which amends and supplements Law 131.13 relating to the practice of medicine and allows foreigners and Moroccans residing abroad to practice medicine in Morocco, and Law no. 39-21 [80], supplements Cherif Dahir no. 1-58-008 by including healthcare professionals working in the public sector on the list of professional categories not covered by this Dahir. This will make it possible to draw up a specific status for these health professionals in order to take better account of the realities of the health system and improve the quality of care in the country. This measure is part of the major changes in the healthcare system in Morocco. Furthermore, the salary grid for early-career physicians, pharmacists, and dentists has been revised, resulting in a significant increase in their starting salaries. The new grid raises the index from 336 to 509 [75].

The government has signed a framework agreement to strengthen training in the healthcare sector. The aim is to train twice as many doctors, pharmacists, and dentists and three times as many nurses and health technicians by 2030 [48]. This initiative will help to meet the growing demand for healthcare professionals, which aims to increase the number of people working in the healthcare sector to 90,000 by 2025, giving a ratio of 24 healthcare professionals per 10,000 inhabitants in 2025, compared with 17.4 currently [81]. This approach aims to reach the minimum threshold set by the WHO of 4.45 doctors, nurses, and midwives per 1,000 inhabitants (44.5 per 10,000) by 2030. This target is in line with the achievement of healthcare-related SDGs, including UHC (SDG 3) and the strengthening of the health workforce (SDG 3.c) [82].

Continuing and professional training: Improved continuing training will enable healthcare professionals to acquire the skills and knowledge necessary to provide quality anesthetic care in all settings and will enable anesthetists to keep abreast of the latest advances and to meet current and future societal expectations and changing standards of healthcare administration [83]. Research has highlighted the importance of continuing training for patient safety, as well as improving the quality of care and the performance of health systems. In terms of continuing training for healthcare professionals, an estimated 24,000 professionals in various specialties benefited from 1,420 training sessions in 2022 [76].

Accreditation: Accreditation is an external evaluation process that ensures the quality and safety of care provided in a healthcare establishment. Independent experts examine the practices and procedures of the establishment to ensure compliance with international standards. Anesthesiology and intensive care have long been attentive to this issue. The French Society of Anesthesia and Intensive Care and the French Language Society of Intensive Care are among the few professional societies that have addressed this problem. The accreditation process they have implemented is conducted by an independent evaluation committee. Its implementation is supported and gradually developed over time [84].

Morocco is committed to achieving accreditation in line with the international quality standards set out in Chapter IX of Framework Law 06.22, which concerns the accreditation system for health establishments. This approach aims to ensure continuous improvement in the quality and safety of care. It provides for an independent assessment of the quality of the services provided by healthcare establishments, based on indicators, criteria, and national benchmarks established by the HAH. The objective is to encourage healthcare establishments to maintain high standards of quality and safety to offer better-quality care to patients. To this end, the MHSP has signed a partnership agreement with Accreditation Canada, the aim of which is to improve the quality and safety of healthcare in Morocco. The agreement provides for Morocco to be accredited according to the international quality standards defined by Accreditation Canada [85].

Outlook for the MHSP

The Moroccan government has taken a crucial step by adopting a major reform of its national healthcare system. This initiative aims to break from outdated models and overcome the current challenges affecting healthcare, particularly anesthetic care. Table 2 provides an enlightening overview of the promising scenarios that this reform can enable for the future of healthcare in Morocco. It highlights the concrete measures taken to remedy the shortcomings of the current system and to meet the ambitious objectives set by the MHSP, as well as international recommendations to create a more equitable, efficient, safe, and sustainable healthcare system.

**Table 2:** Current state, the measures taken, and the desired objectives of the new reform in Morocco CHI: Compulsory Health Insurance; NPR: National Population Register; USR: Unified Social Register; THG: Territorial Health Groups; SDG: Sustainable Development Goal; HAH: High Authority for Health; MAMHP: Moroccan Agency for Medicines and Health Products; MABBP: Moroccan Agency for Blood and Blood Products; MHSP: Ministry of Health and Social Protection; WHO: World Health Organization; CHUs: university hospital centers; CHP: Provincial Hospital Center; LHs: local hospitals: UHC: universal health coverage

Current status	Measure taken	Objective desired
Territorial disparities and unequal access to anesthetic care	Generalize CHI for the entire population and extend affiliation to pension schemes (to include 5 million people by 2025), generalize compensation for loss of employment by 2025, generalize family allowances to 7 million school-aged children (operational in 2023–2024), and put in place operational procedures to generalize the social assistance system by 2024. Implementation of the NPR and the USR [76]. Improving and implementing healthcare provision and infrastructure. The THG is a regional management tool that, under Law 08.22, defines the distribution of medical and paramedical resources across the region [33]. The signing of a partnership agreement to improve access to healthcare in rural areas.	Implement Law 09.21 and extend health insurance to all Moroccans by 2025 [3] to achieve the target 3.8 stated in SDG 3 by 2030, which aims to ensure that everyone benefits from UHC, including protection against financial risks and access to quality essential health services and safe, effective, excellent, and affordable essential medicines and vaccines [69].
Adoption of a new reform of the National Health System	The creation of Law 06.22 [4] for the overhaul of the national health system to ensure: (1) good governance through the creation of HAH by Law 07.22 [34], the THG by Law 08.22 [33], the MAMHP by Law 10.22 [35], and the MABBP by Law 11.22 [36]. (2) The implementation of healthcare services; (3) the development of human resources 09.22 [37]. (4) The digitalization of Chapter VIII of Law 06.22.	Establish a legal framework to reform and restructure the healthcare system in a participatory manner. Promote a preventive healthcare policy, guarantee a balanced supply of care, and strengthen basic care facilities. Be based on up-to-date data and a rational policy.
Lack of financial resources	The budget allocated to the MHSP should be close to MAD30.7 billion, i.e., approximately 7.2% of the general budget of MAD425.1 billion in 2024 [48].	Reach the WHO’s recommendation (12%) and, particularly, that of the African Union's Abuja Declaration (15%) [46]. To achieve target 3.c of SDG 3 significantly increase the health budget and the recruitment, development, training, and retention of health workers in developing countries, particularly in the least developed countries and small island developing states [69].
Shortage of human resources	Adoption of Law 09.22 on the essential guarantees granted to human resources in the health professions, promulgated in 2023 [37]. The number of healthcare professionals in Morocco reached more than 53,000 in 2023, an increase of 37% compared with 2018. This increase was due to the creation of 5,500 new budgetary posts [76]. In 2024, the Finance Bill provides for the allocation of 5,500 additional budgetary posts. In total, between 2017 and 2024, 42,700 budgetary posts have been created, including 35,500 for the Department of Health [48]. Aims to train twice as many doctors, pharmacists, and dentists and three times as many nurses and health technicians by 2030 [48].	Achieve target 3.c of SDG 3: Substantially increase the health budget and the recruitment, development, training, and retention of health personnel in developing countries, particularly in least-developed countries and small island developing states [69]. The signing of a framework agreement aimed at increasing the number of people working in the health sector to 90,000 by 2025 will make it possible to achieve a ratio of 24 healthcare professionals per 10,000 inhabitants by 2025 [81] and continue the aim of reaching the recommended density of 4.45 healthcare professionals per 1,000 population by 2030 [82].
Inadequate offer of care	To reduce inequalities between urban and rural areas and between mountainous and other parts of the country (2017–2023) through the completion of 590 rehabilitation projects and 369 planned construction projects, as well as the acquisition of 735 mobile units [76]. Strengthening hospital care: more than 2,000 additional beds have been acquired, and numerous projects are underway [48]. Major projects include a CHU (Tangiers, 797 beds); a regional hospital center (Rabat, 380 beds); provincial hospitals CHP (Temara, 250 beds); Driouch, 150 beds; LHs (Mhamid, 45 beds; Sidi Youssef Ben Ali Marrakech, 45 beds; Jerf El Melha, 45 beds; Bouskoura, 45 beds); a regional oncology center (Laayoune, 23 beds); and a multidisciplinary clinic (Martil, 34 beds). Other major projects include the Al Hoceima University Hospital (250 beds) and the Ait Ourira LH in the province of Al Haouz (45 beds). Several other projects are also underway, including three CHUs in Rabat [48].	According to the WHO general policy guidelines for strengthening health systems [72] and setting up healthcare offers tailored to the needs of the Moroccan population, Morocco is launching studies for new hospital infrastructure projects, including the following: new CHUs are being planned in the cities of Errachidia, Guelmim, and Beni Mellal, and 52 facilities are under construction. Among the flagship projects on which work has begun are the Provincial Hospital Centre in Berkane and Taourirt and the Regional Hospital Centre in Oujda. Several LHs have also been established in various regions of Morocco, including Ain Ben Mathar Hospital, Tamsalan Hospital, Machraa Bel Ksiri Hospital, Ouled Berhil Hospital, Tafraout Hospital, and Lakhssas Hospital. In 2024, Morocco plans to open several hospitals, including a CHU in Agadir, a CHP in Kénitra, Tarfaya, and Fkih-Ben Salah, several LHs (Tamesna, Figuig, Talsint, Ahfir, and Midar), and psychiatric hospitals in Agadir and Kénitra [48].

## Conclusions

The new reform of the Moroccan healthcare system aims to improve the accessibility, quality, and safety of services and care in general. This reform is particularly important for the field of anesthesiology, which faces several major challenges, such as disparity and inequality in the supply of anesthetic care, a lack of financial resources, a serious shortage of anesthetists, inadequate continuing education, and the need for the appropriate administrative and legal framework regarding current anesthesiology practice.

The success of the reform of the Moroccan health system to improve anesthetic care will depend on the effectiveness of the measures taken and those that are subsequently implemented, such as the development of infrastructure and human resources in anesthesia, to combat territorial disparities and inequalities. Increasing the budget allocated to health, training, and recruiting a sufficient number of anesthetists, adopting an appropriate administrative and legal framework is essential to guarantee patient safety and the quality of anesthesia care, and reinforcing continuous training programs in anesthesia will assist in achieving these goals.

The new reform of the Moroccan health system offers a unique opportunity to improve anesthetic care in the country. The effective implementation of the planned measures will guarantee access to safe, high-quality anesthetic care for all Moroccan citizens. Since the reform is still in its infancy, thorough and rigorous long-term studies will need to be carried out to assess the direct impact of the reform on all aspects of anesthesiology.
